# Prospective data-driven respiratory gating of [^68^Ga]Ga-DOTATOC PET/CT

**DOI:** 10.1186/s13550-021-00775-w

**Published:** 2021-03-31

**Authors:** Jonathan Sigfridsson, Elin Lindström, Victor Iyer, Maria Holstensson, Irina Velikyan, Anders Sundin, Mark Lubberink

**Affiliations:** 1 PET Centre, Uppsala University Hospital, Uppsala, Sweden; 2 Medical Physics, Uppsala University Hospital, Uppsala, Sweden; 3 Nuclear Medicine and PET, Department of Surgical Sciences, Uppsala University, Uppsala, Sweden; 4 Medical Radiation Physics and Nuclear Medicine, Karolinska University Hospital, Huddinge, Stockholm, Sweden; 5 Department of Clinical Science, Intervention and Technology (CLINTEC), Division of Functional Imaging and Technology, Karolinska Institute, Stockholm, Sweden

**Keywords:** PET/CT, DOTATOC, Respiratory gating, Data-driven gating

## Abstract

**Aim:**

The aim of this prospective study was to evaluate a data-driven gating software’s performance, in terms of identifying the respiratory signal, comparing [^68^Ga]Ga-DOTATOC and [^18^F]FDG examinations. In addition, for the [^68^Ga]Ga-DOTATOC examinations, tracer uptake quantitation and liver lesion detectability were assessed.

**Methods:**

Twenty-four patients with confirmed or suspected neuroendocrine tumours underwent whole-body [^68^Ga]Ga-DOTATOC PET/CT examinations. Prospective DDG was applied on all bed positions and respiratory motion correction was triggered automatically when the detected respiratory signal exceeded a certain threshold (*R* value ≥ 15), at which point the scan time for that bed position was doubled. These bed positions were reconstructed with quiescent period gating (QPG), retaining 50% of the total coincidences. A respiratory signal evaluation regarding the software’s efficacy in detecting respiratory motion for [^68^Ga]Ga-DOTATOC was conducted and compared to [^18^F]FDG data. Measurements of SUV_max,_ SUV_mean_, and tumour volume were performed on [^68^Ga]Ga-DOTATOC PET and compared between gated and non-gated images.

**Results:**

The threshold of *R* ≥ 15 was exceeded and gating triggered on mean 2.1 bed positions per examination for [^68^Ga]Ga-DOTATOC as compared to 1.4 for [^18^F]FDG. In total, 34 tumours were evaluated in a quantitative analysis. An increase of 25.3% and 28.1%, respectively, for SUV_max_ (*P* < 0.0001) and SUV_mean_ (*P* < 0.0001), and decrease of 21.1% in tumour volume (*P* < 0.0001) was found when DDG was applied.

**Conclusions:**

High respiratory signal was exclusively detected in bed positions where respiratory motion was expected, indicating reliable performance of the DDG software on [^68^Ga]Ga-DOTATOC PET/CT. DDG yielded significantly higher SUV_max_ and SUV_mean_ values and smaller tumour volumes, as compared to non-gated images.

## Introduction

Respiratory motion has an impact on positron emission tomography (PET) imaging of the thorax and abdomen, usually in the vicinity of the diaphragm. Non-corrected respiratory motion might lead to severe lesion blurring which affects several aspects of the imaging assessment such as delineation, detectability, and quantitation [1, 2]. Application of respiratory gating has proven useful to improve PET image quality [3, 4]. Several different approaches for respiratory gating in PET imaging have been tested and most of them are based on external devices that extract a respiratory signal by tracking the chest wall movements [5]. One common device-based alternative is the Real-time Position Management™ (RPM) Respiratory Gating system (Varian Medical Systems; CA, USA) which records the motion of a box, equipped with reflective markers, placed on the patient’s chest. Another option is provided in AZ-733 V (Anzai Medical Corp., Tokyo, Japan), which uses a pressure belt to register chest motion. However, device-based gating requires extra set-up time near the patient, which increases the radiation dose to staff, and may not always be feasible in the daily routine PET imaging. Also, the chest wall motion may not always correlate fully with that of the abdominal and thoracic organs [6, 7].

Data-driven gating (DDG) constitutes a device-less alternative by which respiratory motion is derived directly from the PET-acquisition data. A number of different techniques for DDG have been developed during the last decade, and many have shown promising results when applied on retrospective data [8–13]. DDG has previously not been available for clinical use, but recently a software based on principal component analysis (PCA) was commercially released, making it possible to apply DDG during the PET acquisition [11, 14]. The respiratory signal generated with PCA-based DDG has been found to correlate well with that from devices, and showed better performance in terms of robustness, image quality, and higher standardized uptake values (SUV). Introduction of DDG into PET clinics might allow for replacement of device-based gating and make routine use of respiratory gating clinically feasible [14–16].

In the present study, the vendor’s integrated software MotionFree (GE Healthcare) was used for DDG. The software applies PCA on a series of 0.5 s frame sinograms to identify periodical motion in the data. The three first principal components are extracted, and fast Fourier transform is applied. A ratio between the signal peak in the frequency span associated with respiratory motion (0.1-0.4 Hz) and the mean signal of the noise (0.4–1.0 Hz) generates a value denoted *R*. The *R* value is a measure of how much the signal resembles respiratory motion in each bed position and hence provides information on to what extent this motion might affect the resulting image. An *R* value threshold can be predefined by the user and during the scanning, when this threshold is exceeded in a bed position, motion correction is triggered automatically. This results in activation of quiescent period gating (QPG), extending the scan time and retaining images from a desired percentage, typically 50%, of the total acquisition. When the level of respiratory signal in a bed position is below the set threshold, it is assumed that there is no need for respiratory gating and the scan time is not extended [14].

To the best of our knowledge, all previously published studies evaluating the MotionFree software’s performance regarding identification of the respiratory signal have been conducted retrospectively on [^18^F]FDG PET data. However, since this DDG software is based on PCA, which is a signal processing method, its sensitivity is expected to depend on noise levels and tracer distribution [17]. We hypothesize that when a high-contrast tracer is used, with high accumulation in a few tissues and low uptake in most tissues, especially a tracer with high liver and spleen uptake, the software will most likely derive a high respiratory signal, thus activating motion correction in more bed positions than with a low-contrast tracer such as [^18^F]FDG.

The somatostatin analogue [^68^Ga]Ga-DOTATOC is used for PET/CT-imaging of neuroendocrine tumours (NETs) [18, 19] and shows uptake characteristics of a high-contrast tracer. The most common types of NET arise from the gastrointestinal and broncho-pulmonary tracts and the most frequent location of distant metastases is in the liver [20, 21]. Consequently, PET/CT imaging with [^68^Ga]Ga-DOTATOC would most likely benefit from respiratory motion correction.

The aim of this prospective study was to evaluate the MotionFree software’s performance, in terms of identifying the respiratory signal, comparing [^68^Ga]Ga-DOTATOC and [^18^F]FDG examinations conducted with the same scanning parameters. In addition, for the [^68^Ga]Ga-DOTATOC examinations, tracer uptake quantitation and liver lesion detectability were assessed.

## Materials and methods

### Production of [^68^Ga]Ga-DOTATOC

The [^68^Ga]Ga-DOTATOC was produced in-house on an automated synthesis platform (Modular PharmLab, Eckert & Ziegler, Germany) using disposable cassette system (C4-Ga68-PP) and pharmaceutical grade ^68^Ge/^68^Ga generator (GalliaPharm®, Eckert & Ziegler). The product was formulated in sterile saline and sterile-filtered in-line. The quality control regarding radiochemical purity, chemical purity and quantity was conducted using high performance liquid chromatography with UV- and radio-detectors connected in series.

### Image acquisition and patients

Twenty-four patients (15 women and 9 men, ages 33–88 years) with confirmed or suspected neuroendocrine tumours underwent whole-body PET/CT examinations with [^68^Ga]Ga-DOTATOC. The examinations were performed at Uppsala University Hospital during a one-year period, from June 2019 to June 2020, when the clinical workflow allowed for additional acquisition time. The study was approved by the regional ethics board for medical research (Dnr 2019/00092) and the need for written consent was waived.

The patients were injected with mean ± SD 1.4 ± 0.2 MBq/kg (range 1.1–2.0) of [^68^Ga]Ga-DOTATOC mean ± SD 64 ± 8 min (range 56–79) prior to the examinations conducted on a Discovery MI digital 4-ring PET/CT system (GE Healthcare) with 198 mm axial field of view. Before each PET-acquisition, a CT was performed in end expiration breath hold for attenuation correction as according to our centre’s standard protocol. The vendor’s integrated software MotionFree (GE Healthcare) was applied prospectively for DDG on all scans and motion correction was triggered automatically when the measured respiratory signal exceeded a threshold set to *R* = 15. The threshold was chosen according to the vendor’s recommendation.

According to the clinical standard acquisition protocol, the scan time was 2 min per bed position, which was automatically prolonged to 4 min by the DDG software for bed positions where the *R* threshold was exceeded. These bed positions were reconstructed with QPG, retaining 50% of the total coincidences. In order to obtain comparable images, i.e. with equivalent count statistics, non-gated images were reconstructed using the first 2 min from each bed position. All images were reconstructed with time-of-flight (TOF) ordered subsets expectation maximization (OSEM) using 3 iterations, 16 subsets, resolution recovery, and a 3-mm Gaussian post-filter. A 17-slice (24%) bed overlap was used. In the following, images reconstructed with QPG are referred to as *gated* images, whereas images without QPG are referred to as *non-gated*.

For comparison of the number of triggered bed positions, [^18^F]FDG PET data from 62 consecutive patients, scanned at Karolinska University Hospital, Huddinge, Stockholm on an identical scanner with the same acquisition time and settings as for the [^68^Ga]Ga-DOTATOC examinations was utilized. The [^18^F]FDG patients were injected with 3 MBq/kg using an autoinjector (MEDRAD® Intego), and the scans were started approximately 60 min post-injection. No further analysis was conducted for [^18^F]FDG PET. The [^18^F]FDG data was merely used to compare the DDG algorithm’s performance in terms of detecting respiratory motion.

### Respiratory signal evaluation

The previously calculated *R* values were extracted from the scanner's database retrospectively for each bed position. The centre of each bed position in relation to that of the liver dome was calculated for every patient. Localization of the liver dome utilized the CT performed for attenuation correction.

Differences in the number of triggered bed positions for [^18^F]FDG and [^68^Ga]Ga-DOTATOC were assessed using a Mann–Whitney test. A *P* < 0.05 was considered statistically significant.

### Quantitative analysis

In non-gated [^68^Ga]Ga-DOTATOC PET-images, well-defined tumour lesions located in bed positions that were triggered by DDG during live acquisition, were assessed for measurements of SUV_max_, SUV_mean_, and volume using auto contour volume of interest (VOI) delineation with 41% of SUV_max_, including up to 5 lesions per patient and a maximum of 3 lesions per organ. The VOIs were transferred to the corresponding gated images. To compare SUV_max_, SUV_mean_ and lesion volume, in gated versus non-gated images, an orthogonal regression was performed. In order to quantify the changes in background activity, a spherical VOI of 16 cm^3^ was placed in normal liver tissue in non-gated images and copied to the corresponding gated images. SUV_mean_ in the spherical liver VOI and lesion-to-liver measures (lesion SUV_max_/liver SUV_mean_) were evaluated. Statistical significance was considered for *P* values below 0.05 using Wilcoxon’s signed rank test. Quantitative measurements were made using an Advantage Workstation Server (AW 3.2 extension 3.0, GE Healthcare). Statistical analyses were performed in Prism 8 (GraphPad Software Inc).

### Lesion detectability

In order to achieve some initial data on the clinical performance of DDG in comparison to the non-gated image sets, lesion detectability was analysed, including all [^68^Ga]Ga-DOTATOC PET/CT examinations in which liver metastases were detected. Liver lesion detectability was evaluated by a senior nuclear medicine physician with 21 years of PET/CT experience. The non-gated and gated PET-image sets of each patient were assessed and compared side by side, and the number of detected metastases was registered. Patients with more than 20 liver metastases were excluded from the comparison. The physician was not blinded to gated versus non-gated image sets.

## Results

### Respiratory signal evaluation

**Fig. 1:**
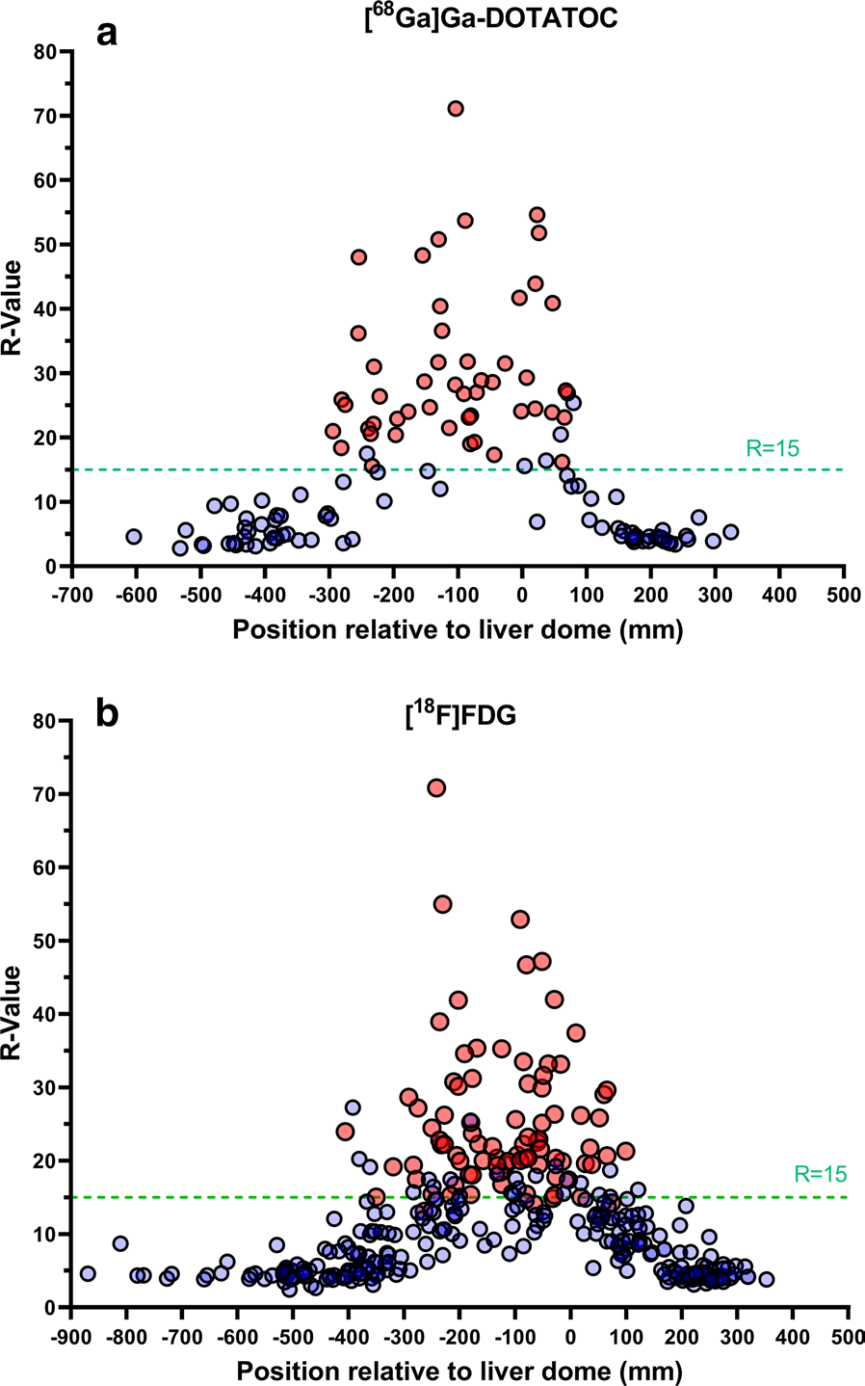
Resulting *R* values for the assessed bed positions, [^68^Ga]Ga-DOTATOC (n = 128) (**a**) and [^18^F]FDG (n = 352) (**b**), as a function of the centre position of the bed position relative to the liver dome. Negative and positive values represent centre positions inferior and superior to the liver dome, respectively. The red dots represent all bed positions that were triggered for motion correction during prospective scanning and the blue dots represent those that were not

A total of 128 bed positions from 24 [^68^Ga]Ga-DOTATOC PET/CT scans and 352 bed positions from 62 [^18^F]FDG PET/CT scans were analysed. There were significantly more triggered bed positions in the [^68^Ga]Ga-DOTATOC PET/CT examinations than in the [^18^F]FDG PET/CT scans (*P* = 0.0074). In the [^68^Ga]Ga-DOTATOC examinations the average number of beds per patient was 5.3. When using a threshold of *R* = 15, QPG was triggered in 22 of the 24 patients and 50/128 bed positions (39%), resulting in mean 2.1 gated bed positions per scan. Bed centre positions in which QPG was triggered were found in the range 7.1 cm superior to 29.5 cm inferior of the liver dome. In the [^18^F]FDG PET/CT examinations, QPG was triggered in 47 of 62 patients and in 87/352 bed positions (25%) using the same threshold (*R* = 15). The number of triggered beds for [^18^F]FDG scans was mean 1.4 and the centre position for the bed positions were found in the range 9.9 cm superior to 40.6 cm inferior of the liver dome (Fig. 1).

### Quantitative analysis

**Fig. 2:**
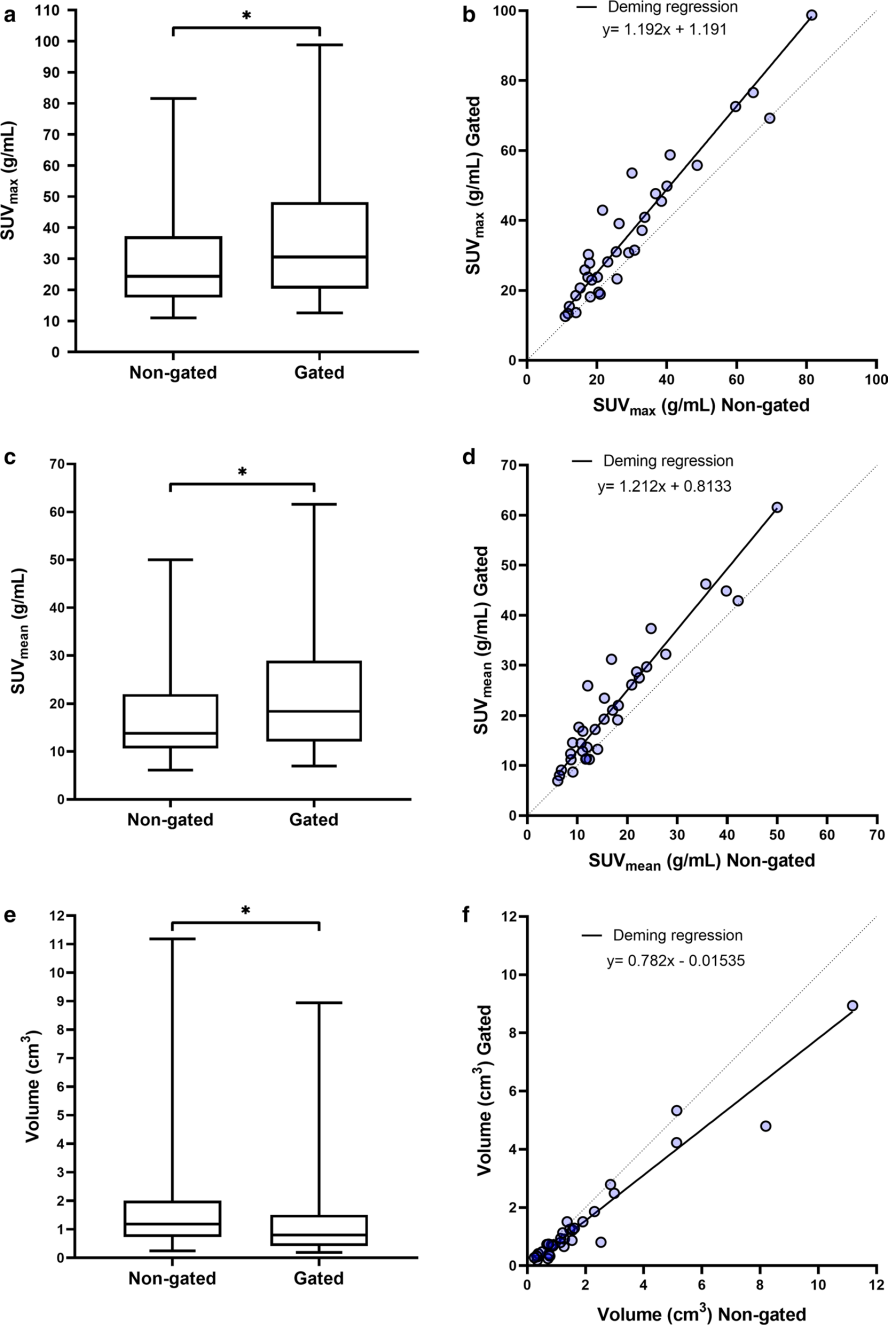
Box plots showing lesion SUV_max_ (**a**), SUV_mean_ (**c**), and volume (**e**) measured in non-gated and gated PET-images. Whiskers show minimum and maximum values and the asterisk indicates *P* < 0.05 using Wilcoxon’s signed rank test. Correlation plots showing lesion SUV_max_ (**b**), SUV_mean_ (**d**), and volume (**f**) of gated versus non-gated PET-images. Solid lines represent linear Deming regression and dotted lines represents line of identity

Of all 24 patients undergoing [^68^Ga]Ga-DOTATOC PET/CT, 14 harboured well-defined lesions in the gated bed positions, which were included for measurements in the quantitative analysis (Table 1). There were 34 measured lesions in total, 17 in the liver, 10 in the intestines, 4 in the lung, and 3 in the pancreas. Figure 2 shows the relation between gated and non-gated SUVs and lesion volumes. By applying DDG, a mean 25.3% increase in the SUV_max_ was achieved, as compared to the non-gated images (*P* < 0.0001). The orthogonal regression analysis, comparing gated and non-gated, of SUV_max_ measurements, resulted in a slope of 1.192 (95% confidence interval (CI) 1.055–1.328). SUV_mean_ was also significantly higher in gated than in non-gated images (28.1%, *P* < 0.0001), with an orthogonal regression slope of 1.212 (95% CI 1.058–1.365). There was a mean 21.1% decrease in lesion volume in gated as compared to non-gated images (*P* < 0.0001), with an orthogonal regression slope of 0.782 (95% CI 0.628–0.937) (Table 2).

**Table 1:** Data from the patients included in the quantitative analysis

Patient #	Gender	Age	Height (cm)	Weight (kg)	Primary tumour location	NET grade	Ki 67	Spread
1	F	44	165	62	SI	2	5–6%	Liver, peritoneum
2	M	68	183	74	SI	1	< 2%	Liver, peritoneum
3	F	67	172	88	SI	1	1.5%	Lymph nodes, liver
4	F	65	172	75	Lung	2	3%	Liver, bone, thyroid, pancreas
5	F	88	158	68	Pancreas	1	1%	Lymph nodes
6	F	67	163	77	Appendix	NA	NA	-
7	M	76	190	80	Rectum	2	14%	Lymph nodes, liver, bone, lung
8	F	68	165	88	SI	1	< 1%	Liver
9	M	75	179	81	SI	1	0,2%	Lymph nodes
10	F	67	164	71	SI	1	1–2%	Lymph nodes, liver, pancreas
11	F	72	162	58	SI	NA	NA	Lymph nodes, liver, bone
12	M	68	180	65	Unknown*	NA	NA	NA
13	M	52	166	64	SI	1	1%	Lymph nodes, liver
14	F	55	170	60	SI	2	18%	Liver, bone

*M* male, *F* female, *NET* neuroendocrine tumour, *SI* small intestine, *NA* not available; *NET with unknown primary location

**Table 2:** Quantitative analysis

	Mean change (%)	*P* value	95% CI
SUV_max_	25.3	< 0.0001	5.5–32.8
SUV_mean_	28.1	< 0.0001	5.8–36.5
Volume	-21.1	< 0.0001	− 37.2 to − 6.3

The mean SUV_mean_ in normal liver tissue was 4.36 (range 2.00–7.70) in non-gated images and 4.37 (range 2.03–7.65) in gated images (*P* = 0.4). The ratio between lesion SUV_max_ and liver SUV_mean_ was increased by 19.6% on average in the gated as compared to non-gated images (*P* < 0.0001).

### Lesion detectability

**Fig. 3:**
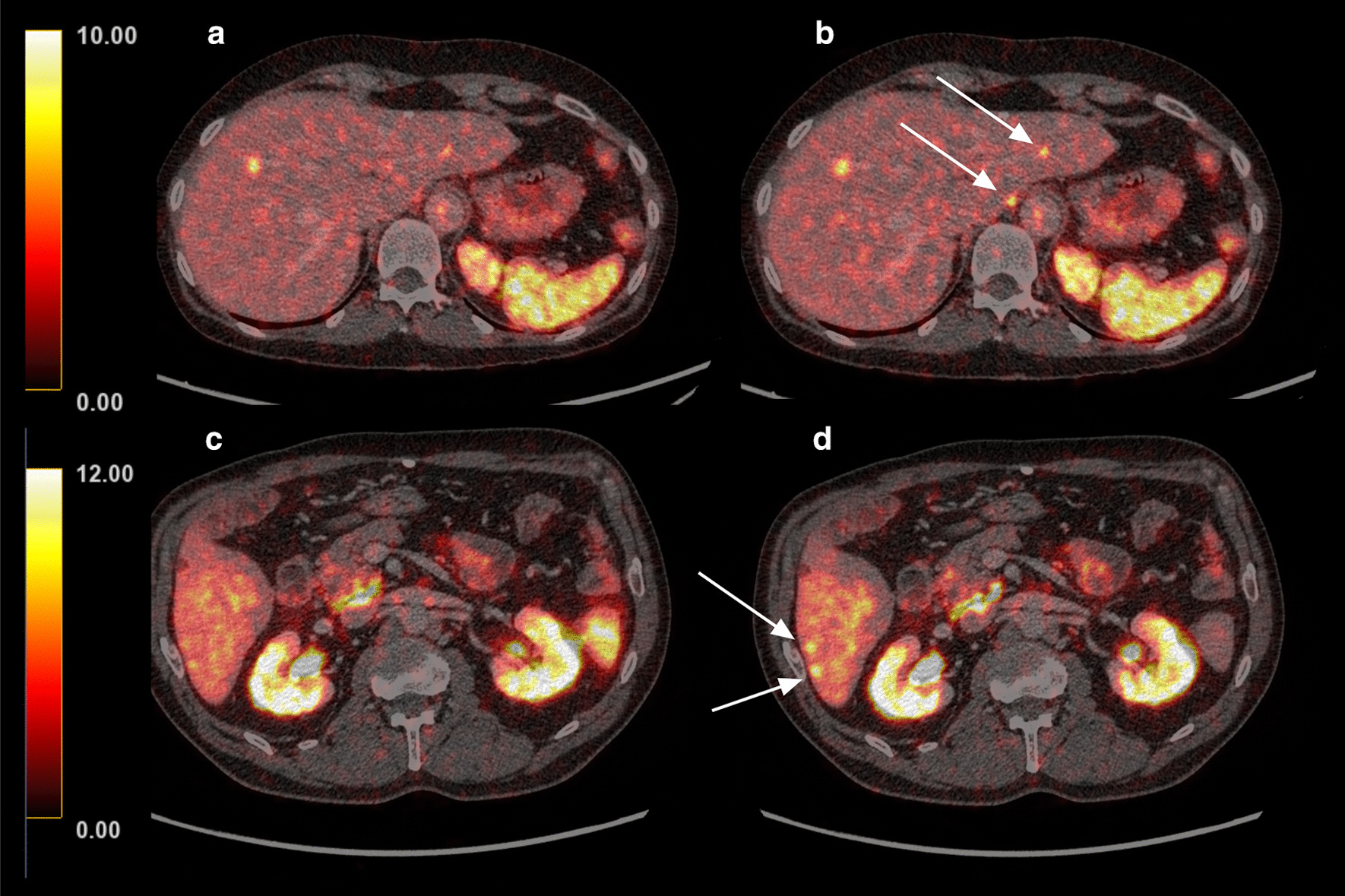
Fused [^68^Ga]Ga-DOTATOC PET/CT images in the transverse plane of two patients with small-intestinal neuroendocrine tumours in the upper panel (**a**, **b**) and in the lower panel (**c**, **d**), respectively. Non-gated images are shown to the left (**a**, **c**) and the gated images to the right (**b**, **d**). In each patient, two additional lesions were diagnosed in the gated image (indicated by arrows)

**Fig. 4:**
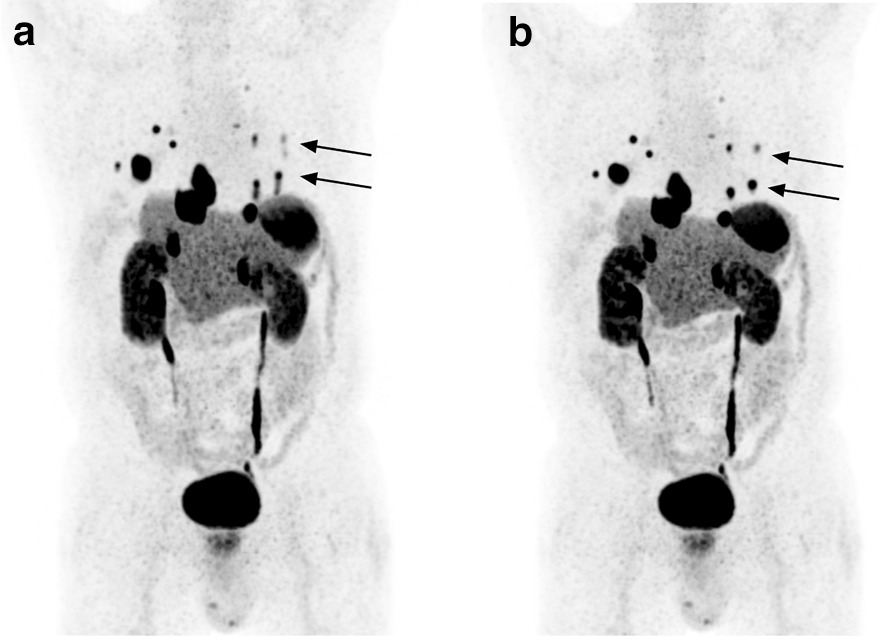
Anterior maximum intensity projections of a non-gated (**a**) and gated (**b**) [^68^Ga]Ga-DOTATOC PET examination in a patient with a rectal neuroendocrine tumour. Multiple lesions in the lung, with large blurring effects caused by respiratory motion are indicated by arrows. Both images have identical SUV_max_ grey scales of 0–15 g/mL

Nine out of the 14 patients in the quantitative analysis harboured liver metastases on [^68^Ga]Ga-DOTATOC PET/CT and were included in a subsequent lesion detectability analysis. Two patients harboured more than 20 lesions and were thus excluded from the analysis. In two patients, 8 and 5 metastases were detected in the non-gated and 10 and 8 in the gated images, respectively (Fig. 3). In five patients, the reader detected an equal number of metastases in the non-gated and gated images. In total, the number of diagnosed liver metastases was 36 in non-gated and 41 in gated images. Generally, the lesions were displayed clearer and more well-defined in the gated images as compared to the non-gated images (Fig. 4).

## Discussion

In this study, several aspects of using DDG on [^68^Ga]Ga-DOTATOC PET/CT were evaluated. The signal from respiratory-like motion in [^68^Ga]Ga-DOTATOC PET/CT was found to originate from areas in the vicinity of the diaphragm, and especially inferior to the liver dome. By using an *R* = 15 threshold, gating was triggered in bed positions where respiratory motion was expected to have a large impact, indicating that DDG reliably detects the respiratory signal in the [^68^Ga]Ga-DOTATOC PET/CT examinations. Because many NET-lesions are located in the vicinity of the diaphragm, especially in the liver, lesion detection is expected to benefit from applying DDG in many [^68^Ga]Ga-DOTATOC PET/CT examinations.

The *R* value threshold of 15 was exceeded in mean 2.1 bed positions per [^68^Ga]Ga-DOTATOC PET/CT scan compared to mean 1.4 for [^18^F]FDG examinations acquired on an identical scanner, showing that the DDG software was more effective in detecting respiratory motion in [^68^Ga]Ga-DOTATOC PET/CT examinations than in [^18^F]FDG PET/CT scans. This is likely due to the higher image contrast properties of [^68^Ga]Ga-DOTATOC. DDG applied on PET/CT examinations with other tracers with similar distribution as [^68^Ga]Ga-DOTATOC, especially other ^68^Ga-DOTA-peptides, may therefore be expected to provide similar results on respiratory signal detection. The axial range in which respiratory motion is detected, however, appears to be similar for [^18^F]FDG and [^68^Ga]Ga-DOTATOC (Fig. 1). DDG used on the [^18^F]FDG PET/CT examinations tended to register less respiratory motion near the diaphragm, thus resulting in a lower overall mean number of bed positions exceeding the *R* threshold. Similar to the present results, a previous study found that DDG applied to [^18^F]FDG PET-scans, acquired on a PET-system with 15 cm instead of 20 cm axial FOV, showed *R* ≥ 15 in mean 1.2 beds per examination [14]. Hence, the axial FOV of the scanner appears to be of minor importance to the sensitivity of DDG in detecting respiratory motion.

One of the most important aspects of respiratory gating is the ability to implement this in clinical routine, which is one of the proposed benefits with DDG as compared to device-based gating. To be able to do so, it is important to predict the expected additional acquisition time, since activation of QPG increases the total scanning time for the PET-examination. In this study, a 2 min /bed acquisition protocol was utilized, and the total acquisition time was prolonged by approximately 3 and 4 min on average for [^18^F]FDG and [^68^Ga]Ga-DOTATOC scans, respectively. Choosing an *R* value threshold is an uncomplicated approach allowing for automatic gating application. However, the efficacy of deriving a respiratory signal will depend on several aspects, even if the same PET-tracer is used. Differences in scanning time, amount of injected activity, respiratory motion constancy and amplitude, etc., will all have an impact in this respect, making it difficult to foresee the optimal *R* value threshold for the individual scan. Altering the *R* value threshold is a way of either increasing or decreasing the probability of gating being triggered. In the current study, most bed positions with low expectancy of being affected by respiratory motion, such as head/neck and upper thighs, showed *R* values ranging from 3 to 6. However, our finding of a few outliers with *R* values around 10, supported that an *R* value threshold ≤ 10 might lead to correction of data in bed positions with limited benefit and consequently unnecessarily prolonged acquisition times. Conversely, an increased *R* value threshold will trigger QPG in fewer bed positions and hence, result in shorter scanning times. Considering the present finding that all bed positions exceeding the respiratory signal threshold corresponded to locations in which respiratory motion was expected, the use of an *R* value threshold higher than 15 would induce a risk for unregistered and uncorrected respiratory motion.

The quantitative analysis showed that SUV_max_ and SUV_mean_ were significantly higher and that tumour volume was significantly lower in gated as compared to in non-gated [^68^Ga]Ga-DOTATOC PET images. The background activity in the liver (SUV_mean_), however, was not significantly different when comparing gated and non-gated images. The NET-patients´ eligibility for peptide receptor radionuclide therapy (PRRT) with ^90^Y and ^177^Lu labelled DOTA-peptides [22, 23] is based on evaluating ^68^Ga-DOTA-somatostatin analogue-PET/CT and comparing the uptake in the tumours to that in reference organs, mainly the liver and spleen [24, 25]. A more accurate quantification of tumour tracer uptake by application of DDG may therefore add to this assessment and make it more reliable.

In order to acquire some initial data on the impact of DDG in a clinical situation, we included also a lesion detectability analysis to our study, although with merely one reader. There was a tendency to depict more liver metastases in the gated than in the non-gated [^68^Ga]Ga-DOTATOC PET examinations. As illustrated by Fig. 4, the metastases were generally sharper delineated in the gated images, consequently facilitating the reading. Introduction of DDG in the clinical [^68^Ga]Ga-DOTATOC PET/CT imaging routine could potentially increase lesion detection and reader confidence. This, however, has yet to be assessed in a separate trial in a larger number of patients, employing several observers and grading reader confidence scores. Also, an evaluation including different image reconstructions, such as Bayesian penalized likelihood reconstructions, could also be valuable since different reconstruction methods applied together with DDG, are likely to impact image quality and PET quantification [26].

Limitations of this study includes that no comparison of DDG versus device-based gating was performed. This has, however, been published previously on retrospective [^18^F]FDG data, in terms of evaluating the respiratory signal, quantitative measurements and image quality, and where the performance of DDG was found superior [15]. Further, the PET/CT findings were not validated by comparison with a standard of reference. However, NET patients with liver metastases rarely undergo liver resection to provide a surgical and histopathological gold standard, although validation of the PET-findings of additional liver metastases could have been achieved by additional imaging, for example magnetic resonance imaging (MRI) including dynamic intravenous contrast-enhancement of the liver, with a hepatocyte-specific contrast medium, and diffusion-weighted MRI sequences. We are because of this unable to prove the increase in detected liver metastases from the lesion detectability analysis. The gated and non-gated images were viewed side-by-side, which is also a limitation, potentially leading to biased results in the lesion detectability analysis. However, based on the promising effects DDG shows on quantitation, and that it requires little effort to implement, we believe that there are adequate indications supporting the introduction of DDG in a clinical setting, and we have now implemented it in our centre as routine practice.

A strength of this study was its prospective nature, which already during the study time provided information on the effects of introducing DDG in the clinical PET/CT-imaging workflow. No additional time or effort was needed before, during or after acquisition for technologists managing the scanner, ensuring a standardized implementation of gating for every patient. In our view, introduction of DDG allows respiratory motion correction to become part of the clinical routine in PET/CT.

## Conclusions

High respiratory signal was exclusively detected in bed positions where respiratory motion was expected, indicating reliable performance of the DDG software on [^68^Ga]Ga-DOTATOC PET/CT and gated images yielded significantly higher lesion SUV and lower lesion volumes as compared to non-gated images. This supports that DDG might be a useful tool to provide more accurate quantitation of [^68^Ga]Ga-DOTATOC tumour uptake.

## Data Availability

The datasets generated during and/or analysed during the current study are available from the corresponding author on reasonable request.
